# Exploring adolescents’ indirect financial and non-financial barriers to dental care non-attendance: the role of payment methods

**DOI:** 10.3389/fpubh.2025.1554171

**Published:** 2025-07-28

**Authors:** Alla T. Alsharif, Saba Kassim

**Affiliations:** Department of Preventive Dental Sciences, College of Dentistry, Taibah University, Al-Madinah, Saudi Arabia

**Keywords:** adolescents, indirect financial, non-financial barriers, dental care, oral health, payment methods

## Abstract

**Background and aims:**

Dental attendance is key to the prevention and early detection of oral diseases. In Saudi Arabia (SA), dental care is publicly funded for citizens; however, many families opt for private care through insurance or out-of-pocket payment. This study has twofold: (1) to examine factors associated with regular dental attendance versus non-dental attendance among adolescents, and (2) to explore the indirect financial and non-financial barriers to dental non-attendance, with a particular emphasis on how payment methods influence these barriers.

**Methods:**

An analytical cross-sectional survey was conducted among a convenience sample of adolescents in Al-Madinah, SA. Data collected included socio-demographic characteristics, oral health related variables (e.g., brushing teeth, dental attendance pattern) and barriers to non-dental attendance. Logistic regression identified factors associated with non-attendance for dental care. To analyse non-dental attendance drivers, we organised response into six thematic domains: affordability, availability, accessibility, motivation, perceived need, and fear/anxiety. We then classified affordability—including transportation costs, productivity loss, and childcare expenses—as an indirect financial barrier, whereas the remaining domains (availability, accessibility, motivation, perceived need, and fear/anxiety) were defined as non-financial barriers. These were compared in relation to the participants’ payment methods.

**Results:**

Among 416 adolescents, (203 males, 48.9%), 315 (75.7%) reported non-dental attendance and 216 (51.9%) used out-of-pocket payment. Regression analysis showed that being male, using publicly funded dental services, and self-rating poor oral health were significantly associated with non-dental attendance (AOR = 2.42; 95% CI: 1.39–4.20; *p* = 0.002; AOR = 2.00; 95% CI: 1.07–3.75; *p* = 0.030; AOR = 2.32; 95% CI: 1.05–5.10; *p* = 0.037, respectively). Indirect financial barriers—such as parental childcare responsibilities and travel costs—were comparable across all payment methods. Fear and anxiety (as non-financial barriers) were more prevalent among adolescents using out-of-pocket or insurance payment methods (*p* = 0.021).

**Conclusion:**

A high rate of adolescents dental non-attendance was significantly associated with demographic, economic factors, as well as with perceived oral health. Both indirect financial and non-financial barriers—except fear and anxiety—were frequently reported among adolescents regardless of payment method. This suggests the need for cost-efficient strategies (e.g., transport support), and psychoeducational approaches to improve dental attendance for both adolescents and their families.

## 1 Introduction

Despite ongoing improvements in overall well-being, disparities in access to dental care remain a global challenge, particularly among vulnerable populations such as adolescents, who continue to suffer from chronic oral conditions and face barriers shaped by social inequality (1). Regular dental visits are essential for preventing and treating dental issues, thereby improving quality of life. Despite public funding for dental care in Saudi Arabia (SA), adolescents are among the least likely to utilise healthcare service. Studies have reported that between 71 and 93.2% of adolescents do not utilise dental services (2–5), highlighting a significant gap in oral healthcare utilisation within this age group. Adolescence is a central period during which health appraisals are being shaped, and behaviors that can either compromise or enhance health are developed (6, 7). These behaviors significantly influence future morbidity and dental healthcare utilisation, as risky behaviors and poor lifestyle choices contribute to many health (7) problems. It is a critical time for health care professionals to intervene before psychological issues and lifestyle habits become embedded by the end of the teenage years. Since patterns of health care utilisation are established early, understanding the predictors of adolescent dental healthcare use can help develop strategies to promote appropriate dental care during adolescence, which can transfer to adulthood.

The literature also indicates that the decision to avoid dental care is typically not intentional but rather a result of various barriers (8, 9). Healthcare system characteristics and social factors are key determinants of access to care. Other factors, as outlined in Andersen et al.’s model of access to healthcare, are equality influential (10, 11). Major barriers to accessing dental treatment include the limited availability of dental services, geographic isolation, fear and anxiety, and various social and economic factors (12). The economic impact of oral diseases is substantial, involving direct treatment expenses, indirect cost such as missed school and workdays, and intangible costs (non-financial) that diminish quality of life (8, 9, 13–16). Although dental care is available for citizens through public funding, many families in SA prefer private dental services, either through insurance or out-of-pocket payment (11).

While previous studies have identified some predictors of adolescent dental healthcare utilisation and applied theoretical framework, such as Andersen’s behavioral model of health service utilisation (17, 18), limited attention has been given in SA to the indirect financial barriers (e.g., arranging childcare or transportation cost) and non-financial barriers (e.g., fear, anxiety, or lack of motivation) that influence non-attendance for dental care, especially across different payment methods, including public funding, insurance, or out-of-pocket payments. The study objectives are: (1) to examine factors associated with regular dental attendance versus non-attendance among adolescents. (2) to explore the indirect financial and non-financial barriers contributing to non-attendance for dental care, with a particular emphasis on how different payment methods influence these barriers. The rational for this research is to inform and influence oral health policies by addressing the indirect financial and non-financial drivers faced by Saudi families with adolescents when seeking dental care.

## 2 Materials and methods

### 2.1 Study design, population, setting and ethical considerations

This analytical cross-sectional of online survey followed STROBE guidelines (19) and was approved (Ref. TUCDRED/20200328/HABakeer) by the Research Ethics Committee of Taibah University, College of Dentistry. The survey adhered to the Declaration of Helsinki (20), including voluntary participation, confidentiality of the information obtained, and the right to withdraw from the survey at any time without providing a reason. A convenience sample of 439 high school children aged 14–18 years, (104 students from private and 335 from public high schools) was recruited in the city of Al-Madinah, SA. Schools and participating students were recruited based on the schools’ consent.

### 2.2 Data collection procedures

The survey was administered in a single day during scheduled school hours. Participating students were guided to a room where they completed an online questionnaire on iPads. The form was accessible via a direct link to a Google Form, using HyperText Markup Language (HTML) and a valid self-administered Arabic questionnaire. Before starting, students received an information sheet about the survey and electronic consent instructions. Only those who provided consent were able to access and complete the questionnaire, which took about 10 min. Upon completion, participants submitted the form, and their responses were stored in an Excel spreadsheet.

### 2.3 Data collection tool and procedures

The survey comprised of validated self-administered questionnaire that included closed ended questions on socio-demographic variables (e.g., age in years, sex, family income (>SR 50,000, SR 40,001–50,000, SR 30,001–40,000, SR 20,001–30,000, SR 10,000–20,000 and <10,000 SR), parents’ educational attainment [postgraduate, university degree (categorised as university or higher), high school, intermediate school, primary school, illiterate (categorised as less than university)], type of school (private or public) and school level (first, second or third high school)]. In addition, oral health related variables included: frequency of tooth brushing per day [categorized as effective oral hygiene (yes) if twice daily, once after waking up and once before sleeping; or as ineffective oral hygiene (No) if once a day or other (e.g., once a week or not at all)]. Payment method for dental treatment (publicly funded, out of pocket payment or dental insurance). Self-rated oral health [“I think my oral health is perfect,” “I think my oral health is very good,” “I think my oral health is good” (categorised as good); “I think my oral health is fair,” “I think my oral health is poor” (categorised as poor)]. Dental attendance (every 3 months, every 6 months, once a year (categorised as regular attendance); or when necessary or never (categorised as non-attendance) (21).

Students reporting non-dental attendance were asked to select from a list more than one answer for their overall non-dental attendance. This list which includes number of drivers for overall non-dental attendance compiled from the literature, e.g., (18, 22, 23). It included the followings: treatment cost and availability, long distance from clinic, transportation cost, difficulties due to my parent’s jobs, I have a responsibility of taking care of my younger siblings, I do not know any good dentist, I do not trust dentists, going to dentists scares me a lot and long waiting list. In addition, participating students were asked to report other drivers. The drivers for non-dental attendance were gathered under different domains (e.g., financial and non-financial).

**Figure 1:**
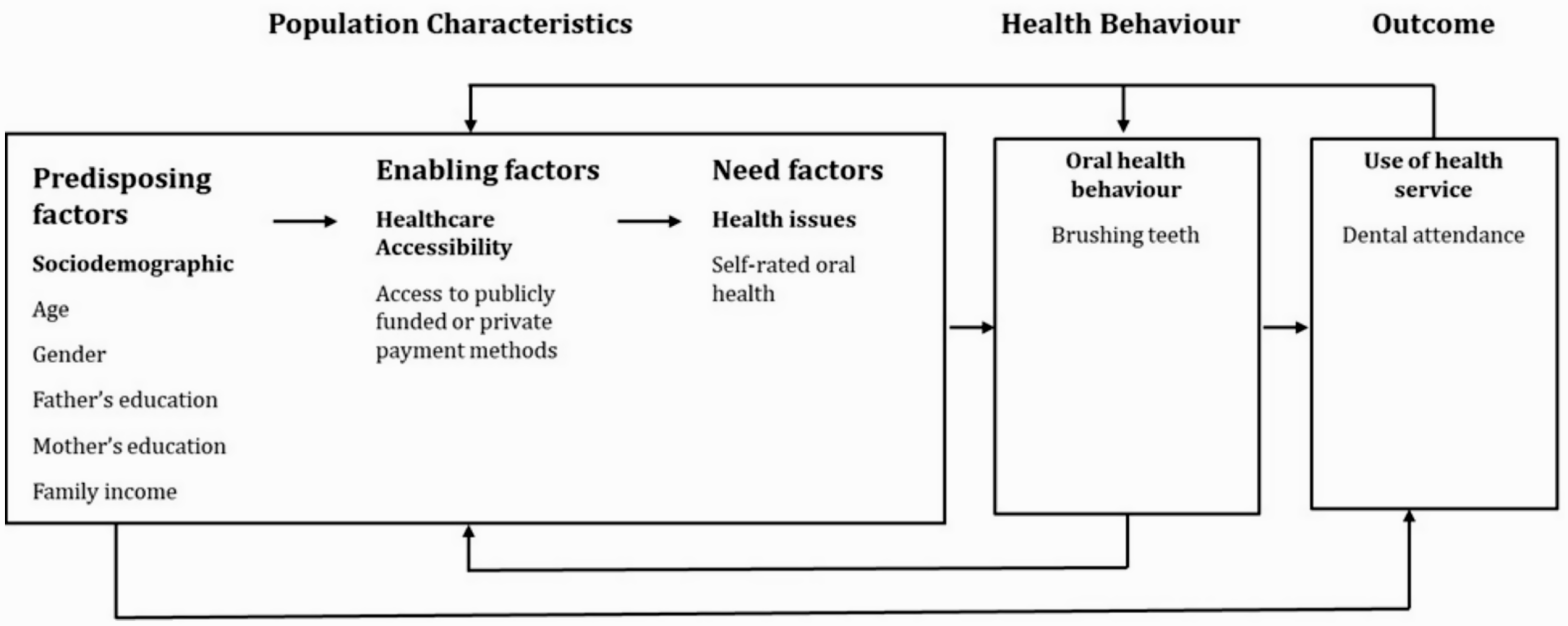
Dental service attendance for Saudi school adolescents, drawn on Gelberg–Andersen Behavioral Model.

This study applies “Andersen’s Behavioral Model of Health Services Use” model (24) to explore the factors influencing dental attendance among adolescents. Andersen’s model provides a framework for understanding healthcare utilisation by examining predisposing factors (such as age, gender, and education level), enabling factors (such as access to publicly funded or private payment methods), behaviors factors (e.g., brushing teeth) and need-related factors (such as self-rated oral health and perceived necessity for care). In our study, these components are analysed to determine their role in non-attendance for dental care (Figure 1). A particular focus of this study on how indirect financial (e.g., transportation costs, childcare responsibilities) and non-financial barriers (e.g., fear, anxiety, lack of motivation) vary across different payment methods is explored. This approach ensures a comprehensive understanding of the personal and general factors influencing healthcare utilisation.

### 2.4 Statistical analysis

All analyses were conducted using the Statistical Package for Social Sciences (SPSS software for Windows, version 21, IBM). Descriptive statistics summarised the study sample and to confirmed test assumption. Categorical variables were reported as frequencies and percentages and the continuous variable (age) as the median and interquartile range (IQR) since the data did not adhere to normality tests (Shapiro–Wilk was <0.05). Some categorical variables were combined to facilitate data interpretation (Table 1 Bivariate analyses (chi-squared and Mann–Whitney U tests) were conducted to assess the association of socio-demographic and oral health related variables with overall non-dental attendance. Students’ high school level (year 1–3) and age were highly correlated; therefore, only age was included in the regression analyses. Parental education level was also entered as confounders.

**Table 1:** Descriptive analysis of sample characteristics and oral health related variables and bivariate analysis of factors associated with non-dental attendance among school adolescents in Al-Madinah, SA, (*n* = 416).

Variable		Total F (%)	Dental attendance F (%)		*p*-Value^a^
			Regular dental attendance (*n* = 101)	Non-dental attendance (*n* = 315)	
Age in years, median (IQR)		16 (1)	17 (1)	16 (1)	**0.017** ^b^
Sex	Female	213 (51.2)	69 (32.4)	144 (67.6)	**<0.001**
	Male	203 (48.8)	32 (15.8)	171 (84.2)	
Type of school	Private	319 (76.6)	84 (26.3)	235 (73.7)	0.076
	Public	97 (23.3)	17 (17.5)	80 (82.5)	
Student’s high school level	First year	209 (50.2)	39 (18.7)	170 (81.3)	**0.007**
	Second year	127 (30.5)	33 (26.0)	94 (76.0)	
	Third year	80 (19.2)	29 (36.2)	51 (63.8)	
Father’s level of education	University or higher	274 (65.9)	62 (22.6)	212 (77.4)	0.275
	Less than university	142 (34.1)	39 (27.5)	103 (72.5)	
Mother’s level of education	University or higher	230 (76.9)	80 (25.0)	240 (75.0)	0.531
	Less than university	96 (23.1)	21 (21.9)	75 (78.1)	
Family income	High (more than 20,000 SR)	176 (42.3)	43 (24.4)	133 (73.6)	0.155
	Middle (10,001–20,000 SR)	148 (35.6)	42 (28.4)	106 (71.6)	
	Low (10,000 and less SR)	92 (22.1)	16 (17.4)	76 (82.6)	
Dental treatment payment#	Out of-pocket	216 (51.9)	66 (30.6)	105 (69.4)	**0.007**
	Dental insurance	83 (20.0)	19 (22.9)	64 (77.1)	
	Publicly funded	109 (26.2)	16 (14.7)	93 (85.3)	
Brushing teeth Twice a day (W. up and B. S)^c^	Yes	241 (57.9)	67(27.8)	174 (72.2)	**0.049**
	No	175 (42.1)	34 (19.4)	141 (80.6)	
Self-rated oral health	Good	343 (82.5)	92 (26.8)	215 (73.2)	**0.009**
	Poor	73 (17.5)	9 (12.3)	64 (87.7)	

a *p*-Value chi-square tests for all analyses unless specified.

b Mann Whitney U, **#**missing data (8 participants).

c Wake up and before sleeping; data is presented as frequency (%) unless specified.

Bold values signify (*p* < 0.05).

A hierarchical multivariable logistic regression modelling was performed, based on the proposed theoretical background (Figure 1), to identify factors associated at *p* ≤ 0.05 with non-dental attendance. Multicollinearity was assessed by examining Variance Inflation Factor (VIF) values for all independent variables and all were <5.0 (range 1.03–1.38), indicating that multicollinearity was not a concern for the logistic regression model. The Hosmer-Lemeshow goodness-of-fit test indicated that the model adequately fit the data, Chi-square value with degree of freedom *χ*^2^(df) = 2.84 (8), *p* = 0.943.

Results are presented as adjusted odds ratios (AORs) with 95% confidence intervals (CIs). As *a priori* sample size was not calculated for this survey, *post hoc* sample calculation showed sufficient power for performing the logistic regression modelling (*R*^2^ = 0.230, predictors = 7, *p* ≤ 0.05 and observed statistical power = 1.0). The *p* ≤ 0.05 was considered significant for all analyses. To analyse non-dental attendance drivers, we extracted responses to the multiple-response question (Supplementary file 1, Q11) and organised them into six thematic domains: affordability, availability, accessibility, motivation, perceived need, and fear/anxiety. We then classified affordability—including transportation costs, productivity loss, and childcare expenses—as an indirect financial barrier, whereas the remaining domains (availability, accessibility, motivation, perceived need, and fear/anxiety) were defined as non-financial barriers. For each of these six domains, a binary variable was created, indicating whether a respondent reported any driver within that domain (1 = Yes, 0 = No). These six dichotomous domain variables were then cross-tabulated with the primary method of treatment payment (publicly funded, out-of-pocket, or dental insurance), and Chi-square tests were performed to explore any significant associations (Table 2).

**Table 2:** Drivers of non-dental attendance (problem-based attendance or never go dentist) among adolescents with different dental treatment payment in Al-Madinah, SA, (*n* = 416).

Drivers for all-over non-dental treatment	Public (yes) F (%)	Out-of-pocket and dental insurance (yes) F (%)	*p*-Value^a^
Availability	32 (34.4)	73 (34.1)	0.960
Accessibility	19 (20.4)	36 (16.8)	0.449
Affordability	23 (24.7)	50 (23.4)	0.796
Fear and anxiety	23 (24.7)	82 (38.3)	**0.021**
Perceived need	5 (5.4)	18 (8.4)	0.353
Motivation	15 (16.1)	23 (10.7)	0.188

a Chi-squared, Bold values signify *p* ≤ 0.05.

## 3 Results

### 3.1 Socio-demographic and oral-health related characteristics

Of the recruited adolescents (439), seven declined participation (98.4% response rate), and 16 were excluded as they were not Saudis (temporary residency), leaving Usable 416 for analysis. The median age was 16 years; 203 (48.8%) were male and 176 (42.3%) from high income families. As for oral health related variables 241 (57.9%) brushed their teeth when they wake up and before sleeping and 343 (82.5%) rated their oral health as good. Table 1 shows other characteristics.

### 3.2 Bivariate analysis of factors associated with non-dental attendance

Regular attendee (every 3 months, every 6 months, once a year) were 101(24.3%) and the remainder (when in pain or never go) 315 (75.7%) reported non-dental attendance (Table 1). Factors significantly associated with non-dental attendance included younger age (*p* = 0.017), being male (*p* < 0.001), first-year high school status (*p* = 0.007), use of publicly funded treatment (*p* = 0.007), brushing teeth less than twice daily (wake up and before Sleeping) (*p* = 0.049) and self-rated oral health as poor (*p* = 0.009).

Table 3 present the regression model results indicating significant factors associated with overall non-dental attendance. Male students and those receiving publicly funded dental treatment were statistically significantly [adjusted odds ratio (AOR) = 2.42 (95% Confidence interval (CI): 1.39–4.20; *p* = 0.002); AOR = 2.00 (95% CI, 1.07–3.75, *p* = 0.030), respectively] more likely to report non-dental attendance than female students and those with other payment methods. In addition, adolescents who self-reported poor oral health were 2.32 times more likely (95% CI, 1.05–5.10, *p* = 0.037) to report non-dental attendance compared with those reporting good oral health.

**Table 3:** Results of adjusted hierarchical multivariable logistic regression analyses predicting the likelihood of overall none-dental attendance among high school adolescents in Al-Madinah, SA, (*n* = 416).

Variable	Model 1			Model 2			Model 3			Model 4		
	Predisposing factors (PF)			PF and enabling factors (EF)			PF, EF and self-reported needs factors (SRNF)			PF, EF, SRNF and behavioral factors		
	B	AOR (95% CI)	*p*-Value	B	AOR (95% CI)	*p*-Value	B	AOR (95% CI)	*p*-Value	B	AOR (95% CI)	*p*-Value
Age	−0.216	0.81 (0.64–1.02)	0.068	−0.200	0.82 (0.65–1.04)	0.094	−0.175	0.84 (0.66–1.06)	0.147	−0.174	0.84 (0.66–1.07)	0.151
Sex			**<0.001**									**0.002**
Female		**Ref.**			**Ref.**	**0.001**	0.812	**Ref.**	**0.001**		**Ref.**	
Male	0.868	2.38 (1.47–3.86)		0.831	2.30 (1.41–3.75)			2.25 (1.38–3.69)		0.883	2.42 (1.39–4.20)	
Father’s level of education			0.501						0.527			0.536
University or higher		**Ref.**			**Ref.**	0.569		**Ref.**			**Ref**	
Less than university	−0.166	0.85 (0.52–1.374)		−0.144	0.87 (0.53–1.42)		−0.161	0.85 (0.52–1.40)		−0.158	0.85 (0.52–1.41)	
Mother’s level of education			0.545			0.646			0.847			0.830
University or higher		**Ref.**			**Ref**			**Ref**			**Ref.**	
Less than university	0.176	1.19 (0.68–2.10)		0.136	1.15 (0.64–2.04)		0.058	1.06 (0.59–1.91)		0.065	1.07 (0.59–1.92)	
Dental treatment payment												
Out-of-pocket					**Ref.**			**Ref.**			**Ref.**	
Dental insurance				0.356	1.43 (0.78–2.62)	0.250	0.343	1.41 (0.77–2.60)	0.271	0.343	1.41 (0.77–2.60)	0.271
Publicly funded				0.743	2.10 (1.13–3.91)	**0.019**	0.682	1.99 (1.06–3.70)	**0.033**	0.694	2.00 (1.07–3.75)	**0.030**
Self-rated oral health									**0.045**			0.**037**
Good								**Ref.**			**Ref.**	
Poor							0.781	2.18 (1.02–4.68)		0.840	2.32 (1.05–5.10)	
Brushing teeth										−0.169		0.559
Twice a day (W. up and B. S)^a^											**Ref.**	
Other^b^											0.84 (0.48–1.49)	

a W. up and B. S = wake up and before sleeping.

b Other = e.g., once daily or weekly; AOR: adjusted odds ratio; CI: confidence interval, Bold values signify *p* ≤ 0.05.

Regarding the drivers of overall non-dental attendance, Table 2 indicates that students reported comparable rates of barriers related to availability, accessibility, affordability, low perceived need, and lack of motivation (*p* > 0.05). Fear and anxiety, however, were significantly more likely (*p* < 0.05) to be reported as a dental attendance barrier by adolescents using out-of-pocket or dental insurance payment methods.

## 4 Discussion

This study examined factors associated with regular dental attendance versus non-dental attendance among adolescents in Al-Madinah, SA. The overall non-dental attendance rate was high, with more than 75% of participants only attending only for pain or never attending at all. This finding aligns with previous studies (2–5).

A logistic regression analysis identified that being male, using publicly funded dental services, and self-reported poor oral health were significant predictors of non-dental attendance among adolescents. Male adolescents in Saudi report non-attendance for dental care more often than females, possibly due to gender-specific health-seeking behaviors and cultural norms. Studies indicate that societal norms associating masculinity with independence and self-sufficiency may discourage males from seeking regular healthcare (25). In contrast, females demonstrate higher health literacy and engaged more in preventive health behaviors, influenced by targeted awareness program and cultural expectations. This aligns with Anderson’s model, which identifies demographic characteristics, such as gender, as predisposing factors influencing healthcare behavior (26). Implementing educational programs targeting males, that consider underlying social and behavioral factors (27), could narrow this gap.

Moreover, adolescents relying on publicly funded dental services reported significantly higher non-dental attendance compared to those using out-of-pocket or insurance payment methods as aligned with literature (22). This reflects enabling factors in Andersen’s model, where access to resources (e.g., payment methods) impact service utilization. This may indicate potential challenges in accessibility or quality perceptions associated with publicly funded care, contributing to an over-reliance on emergency-based visits rather than preventive care. Furthermore, efforts to improve the accessibility and quality of publicly funded dental services could encourage greater engagement with preventive care.

Furthermore, those who reported poor self-rated oral health were more than twice as likely to avoid dental visits, supporting the role of perceived health status in driving healthcare behavior (28, 29). Interestingly, neither parental education level nor family income were statistically significant associated with dental attendance, indicating that other factors, such as psychosocial and motivation may play a more decisive role in attendance patterns (30). Notably, Schools and community programs can play a critical role in promoting positive oral health behaviors and changing students’ perceptions of the importance of routine dental care.

Interestingly, neither parental education nor household income was significantly associated with attendance suggests that, once basic financial barriers are removed through universal public coverage, adolescents’ care-seeking behaviors are influenced more by socio-cultural dynamics than economic resources. Strong cultural expectations grant adolescents substantial autonomy in health decision-making, so factors like peer norms, perceived stigma, and individual health beliefs become the primary determinants of preventive care-seeking (31).

Our study also explored indirect financial and non-financial barriers contributing to non-attendance for dental care, with a particular emphasis on understanding how various payment methods influence these barriers. Fear and anxiety emerged as significant limitations for non-dental attendance, especially among adolescents paying out-of-pocket or through insurance. This suggests that, even when adolescents can afford dental care, emotional experience may act as a barrier to seeking treatment and could potentially limit preventive visits (32, 33). Addressing fear and anxiety through patient-centered approaches, such as anxiety management interventions or adolescent-friendly dental services, could reduce these emotional barriers. Other barriers, such as availability, affordability, and perceived need, were similar across different payment methods, indicating that financial factors alone do not fully explain the non-attendance behavior.

The present study has several strengths that included the use of validated questions (21), the use of school setting which is aligned with ‘Healthy Schools” definition that meet the global standards, policies and procedures of World Health Organisation (34), in addition, to launched National Transformation Programme 2020 and Saudi vision 2030, in terms of education and health aspects (35). There are limitations that should be considered when interpreting the results. Its cross-sectional study limits the ability to establish causality. The convenience sample precludes generalizability of the findings and school type with a higher proportion of adolescents from public school may introduce bias. The self-report of number of variables without objective validation may introduce recall bias, though such an approach is generally used in epidemiological studies, in addition, to social desirability bias. Future research using clinically assessed oral health data, integrated with longitudinal analyses and causal inference methods, could provide greater understanding of how dental visiting patterns may be influenced.

To translate these findings into practice, we recommend integrating oral health screening and preventive services directly within schools to identify at-risk adolescents early and reduce reliance on emergency care. Simultaneously, operating mobile dental clinics across underserved communities can overcome transportation barriers, extend service hours, and ensure continuity of care. Lastly, establishing partnerships with community youth centers will harness existing social networks to provide coordinated transportation assistance, targeted oral health education, and anxiety-reduction workshops—addressing both financial and emotional obstacles to regular dental attendance.

## 5 Conclusion

The study highlighted the complex relationship between socio-demographic, behavioral, and emotional factors (e.g., fear and anxiety) influencing dental attendance patterns. High non-dental attendance among adolescent in Al-Madinah was associated with being male, relying on publicly funded care and having a negative perception oral health. The shared drivers of non-dental attendance among this group, regardless of treatment payment plan, were highlighted. The findings suggest the need for cost-efficient strategies (e.g., transport support), and psychoeducational approaches to improve attendance for both adolescents and families. Future studies should systematically explore interaction effects between key demographic factors (e.g., age, gender), payment methods, and barrier domains (e.g., fear/anxiety) to clarify their combined influence on adolescent dental non-attendance.

## Data Availability

The datasets presented in this study can be found in online repositories. The names of the repository/repositories and accession number(s) can be found below: The data presented in this study are available on reasonable request from the first author.
