# Supplementary material for: Exploring adolescents’ indirect financial and non-financial barriers to dental care non-attendance: the role of payment methods
Source: Front Public Health. 2025 Jul 28;13:1554171. doi: 10.3389/fpubh.2025.1554171 (PMC12336191; doi:10.3389/fpubh.2025.1554171)
Supplement: Supplementary file 1 [file Supplementary_file_1.docx]

**Oral and Dental health - 2021**

Dear high-school student,

We are a group of students from Taibah University Collage of Dentistry who are conducting an Oral health promotion program. You are invited to participate in this study which is concerned with the oral health level in Almadina, Saudi Arabia.

If you agree to be in this study, you will be asked to complete an anonymous questionnaire that will take few minutes to complete. Completion of the questionnaire will be indicative of your consent to participate. The questionnaire will take around seven to ten minutes to answer the questionnaire.

Participating in the study is voluntary and you are still free to withdraw at any time and without giving a reason. Choosing to either take part or not will have no impact on you. All information collected will be kept strictly confidential.

This questionnaire was approved by Scientific Research Ethics Committee at Taibah University and you can contact the principal researcher for any inquiries or complaints:

Hbakeer@taibahu.edu.sa

We appreciate your cooperation

Participation approval

I acknowledge reading the previous information and I would like to participate in this questionnaire.

- [ ] I would like to participate

- [ ] I would not like to participate

School name ..............................

Class name ...........................................................................................

**Personal information:**

1.Sex


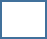
 Male
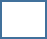
 Female

2.Age


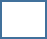

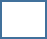


3.Nationality


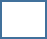
 Saudi
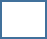
 Non-Saudi

4.Your marital status:


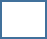
 Single
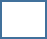
 Married
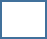
 Separated

5. The educational level of the father:


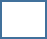
 Illiterate
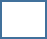
 Elementary
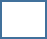
 Middle school
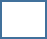
 High school

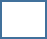
 University graduate
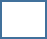
 Postgraduate

6. The educational level of the mother:


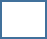
 Illiterate
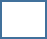
 Elementary
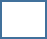
 Middle school
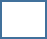
 High school

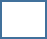
 University graduate
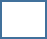
 Postgraduate

7. The family average monthly income approximately:


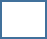
 Less than SR 3000


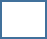
 SR 3000 to SR 10,000


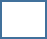
 SR 10,001 to SR 20,000


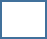
 SR 20,001 to SR 30,000


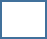
 SR 30,001 to SR 40,000


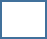
 SR 40,001 to SR 50,000


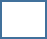
 More than SR 50,000

**Questions on oral health:**

8. How many times per day do you brush your teeth?


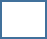
 I don't brush my teeth


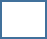
 One time when I wake up


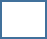
 One time before I sleep


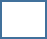
 Twice a day (before sleeping and when waking up)


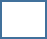
 More twice a day

9. How often do you go to the dentist?


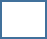
 Every three months
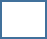
 Every six months
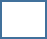
 Once a year


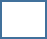
 When necessary
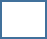
 I do not go

10. How do you rate your oral health status?


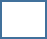
 I think my oral health is perfect.


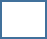
 I think my oral health is very good.


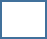
 I think my oral health is good.


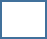
 I think my oral health is fair.


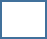
 I think my oral health is bad.

11. Read the following statements and choose the most appropriate options

(you can choose more than one)

**The reasons preventing me from obtaining needed dental care**


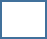
 Treatment cost


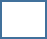
 The long distance from clinic


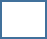
 Transportation cost


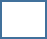
 Difficulties due to my parent's jobs


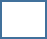
 I have a responsibility of taking care of my younger siblings


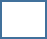
 I do not know any good dentists


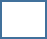
 I do not trust dentists


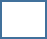
 Going to dentists scares me a lot


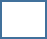
 Long waiting list

Others ……………………………………………………………….

Thanks for your participation
